# Comparison of Breast Cancer-Related Lymphedema (Upper Limb Swelling) Prevalence Estimated Using Objective and Subjective Criteria and Relationship with Quality of Life

**DOI:** 10.1155/2013/807569

**Published:** 2013-06-18

**Authors:** Catherine Bulley, Susanne Gaal, Fiona Coutts, Christine Blyth, Wilma Jack, Udi Chetty, Matthew Barber, Chee-Wee Tan

**Affiliations:** ^1^School of Health Sciences, Queen Margaret University, Edinburgh, Queen Margaret University Drive, Musselburgh, East Lothian EH21 6UU, UK; ^2^Breast Unit, Western General Hospital, Crewe Road South, Edinburgh EH4 2XU, UK

## Abstract

This study aimed to investigate lymphedema prevalence using three different measurement/diagnostic criterion combinations and explore the relationship between lymphedema and quality of life for each, to provide evaluation of rehabilitation. Cross-sectional data from 617 women attending review appointments after completing surgery, chemotherapy, and radiotherapy included the Morbidity Screening Tool (MST; criterion: yes to lymphedema); Lymphedema and Breast Cancer Questionnaire (LBCQ; criterion: yes to heaviness and/or swelling); percentage limb volume difference (perometer: %LVD; criterion: 10%+ difference); and the Functional Assessment of Cancer Therapy breast cancer-specific quality of life tool (FACT B+4). Perometry measurements were conducted in a clinic room. Between 341 and 577 participants provided sufficient data for each analysis, with mean age varying from 60 to 62 (SD 9.95–10.03) and median months after treatment from 49 to 51. Lymphedema prevalence varied from 26.2% for perometry %LVD to 20.5% for the MST and 23.9% for the LBCQ; differences were not significant. Limits of agreement analysis between %LVD and the subjective measures showed little consistency, while moderate consistency resulted between the subjective measures. Quality of life differed significantly for women with and without lymphedema only when subjective measurements were used. Results suggest that subjective and objective tools investigate different aspects of lymphedema.

## 1. Introduction

Progressions in the treatment of breast cancer have led to increased survival rates and increased emphasis on improving outcomes and quality of life in the long term through targeted rehabilitation. Lymphedema is a condition that can develop early or years after treatment, due to the necessary rigor of surgery and radiotherapy which can interrupt the lymphatic system to varying degrees depending on the type of surgery and the dose of radiotherapy [1]. As a result, interstitial tissue can accumulate in the tissues, leading to skin fibrosis and cellulitis. There is evidence of substantial impacts of lymphedema on quality of life and functional outcomes [2]. There is evidence to support rehabilitation interventions specific to the management of lymphedema as a long-term condition, which are summarized in an international consensus document; they include skin care, specialized massage, sustained compression, and exercise [3]. When lymphedema is detected early, therapeutic management is more likely to be effective [4]. 

The incidence of lymphedema is likely to be affected by the increasing use of less conservative surgical techniques for breast cancer, such as breast-conserving surgery and sentinel lymph node biopsy, although this may also increase the use of axillary radiotherapy [5]. Researchers have investigated different possible risk factors for the development of lymphedema; a systematic review of prevalence and risk factors found the former to range from 0 to 34%. It established a pooled odds ratio for developing lymphedema of 1.46 (95% CI 1.16–1.84; 8 studies) for patients who have received radiotherapy compared to those who have not [6]. No differences were found in risk when comparing mastectomy with breast-conserving surgery in only three studies, but lymphedema was 11.67 times more likely (95% CI 1.45–93.65; 3 studies) where full axillary lymph node dissection (ALND) was conducted, compared with sentinel lymph node biopsy (SLNB). In one cross-sectional study comparing these two surgical procedures lymphedema prevalence associated with ALND was 43.3% and with SLNB was 22.2% (*n* = 102) [7]. A prospective study of 936 women evaluated five years after surgery found self-reported arm swelling in 3% of patients after SLNB and 27% with SLNB/ALND and objectively measured lymphedema of 5% and 16%, respectively [8]. Although prevalence estimates vary, it appears that SLNB poses a lower risk of lymphedema, supporting its use. 

In order to assist developments in breast cancer treatment and subsequent rehabilitation, it is very important to estimate prevalence after different types of treatment and to monitor lymphedema over time. However, currently there is wide variability in prevalence estimates, from 2 to 86%, resulting from study samples with different treatment characteristics, the use of different measurements, and varied diagnostic criteria [5]. 

When considering measurement of lymphedema, most objective tools focus on limb volume. Volumetry (water displacement) was considered the gold standard until fairly recently but was not practical clinically, and instead, circumferential measurement of limb segments was used, alongside geometric formulae to enable estimation of volume. More recently, however, perometry (opto-electronic scanning) and multifrequency bioelectrical impedance measurements have gained in popularity as they are more reliable, fast to conduct, and hygienic [1, 9]. When using this form of tool, diagnosis of lymphedema ideally relies on comparisons between pre- and postsurgical measurements, although in cross-sectional studies bilateral limb comparisons are usually made. Differences seen to indicate lymphedema vary, most commonly stated to be equal to or greater than 10% or 200 mL limb volume or 2 cm or 5 cm differences in limb circumference [10, 11]. 

There is evidence to suggest that subjective assessment through patient self-report is more sensitive to the development of lymphedema, as well as being less expensive [1, 5, 11]. Diagnosis of the condition usually focuses on the presence of specific symptoms, such as “heaviness” or “swelling.” They also address functional and psychosocial aspects of the condition rather than focusing on physical dimensions. A combined approach is recommended by some [1]. 

Some studies report good correlations between measurement methods such as circumferential and volumetry measurements, but these do not agree and cannot be used interchangeably. They also focus on measurement of variables such as limb volume rather than estimation of incidence or prevalence [12, 13]. 

One study demonstrated the impact of the classification or diagnostic criterion used with a single objective measure; sequential arm circumference measurement in 347 women was conducted twelve months after surgery [14]. Prevalence of 11% was found within the sample when lymphedema was defined as 2 cm or greater difference between limbs at any measurement point. Limb volume was calculated based on a truncated cone formula, and prevalence differed when using the criteria of 150 mL or greater difference between limbs (9%) and 5% or greater (16%). 

A small number of studies have compared prevalence of lymphedema within samples estimated using different tools and diagnostic criteria. One study compared limb volume changes before surgery to those 12 months after diagnosis in 118 participants, using perometry, circumferential measurement, and self-report [11]. Twelve-month prevalence estimates varied from 21% to 70% prevalence, with perometry measurement (10% or greater change in limb volume) found to be the most conservative and 2 cm difference in limb circumference most sensitive. The same research group followed up 211 women 2.5 years after treatment and again found 2 cm difference in circumferences to be the most sensitive (91%) but found self-report to be the most conservative (41%) [15]. One further study of 176 women found higher estimation by self-report (27.9%) than by objective criteria, which varied between 0.6% (10% or greater difference in summed arm circumference), 11.4% (multifrequency bioimpedance: 3 or more SD above the reference score), and 11.9% (5 cm or greater difference in summed arm circumference) [10]. Therefore, the literature does not demonstrate consensus regarding whether subjective or objective measures provide higher estimates of lymphedema prevalence, or the most valid.

With no gold standard for measuring and classifying lymphedema, it is difficult to know which method produces the most valid prevalence estimate. One way of investigating this is to look at how a construct expected to vary with lymphedema incidence, such as quality of life, relates to estimates provided by different systems of measuring and classifying lymphedema. Indicators of quality of life, psychological morbidity, depression and anxiety, and quality of life scores have been found to differentiate between women who have and do not have lymphedema after breast cancer treatment [16, 17]. One study found that lymphedema was the strongest of three factors that were independently associated with quality of life after treatment for breast cancer, along with nonwhite race and postmenopausal status [18]. 

To conclude, it is important to understand the relationship between subjective and objective estimations of lymphedema prevalence, to make future decisions relating to choice of measurement tool and classification of lymphedema. This secondary data analysis addresses four main questions: how do three different systems of measuring and classifying lymphedema differ in their estimation of prevalence?

- Do the three different systems of measuring and classifying lymphedema identify the same subsample of subjects as having lymphedema?
- What are the sensitivity and specificity of the self-reported systems in relation to the objective measure (perometry) and classification of lymphedema?
- Does the relationship of self-reported quality of life with lymphedema differ when measured with three different systems of measuring and classifying lymphedema?

## 2. Methods

### 2.1. Study Design

This paper represents secondary analysis of a dataset from a cross-sectional study which aimed to screen for fatigue, impaired upper limb function, lymphedema, and pain in women following treatment for breast cancer. It was classified as service review by the South East Scotland Research Ethics Committee and full ethical review was carried out within the Higher Education Institution to ensure that procedures were in accordance with their standards (Declaration of Helsinki 1975, revised Hong Kong 1989). 

### 2.2. Procedure

Subjective and objective measurements of morbidity and quality of life were carried out on women who provided informed consent to participate while awaiting review appointments at the Breast Clinic. Women were approached with information and consent form if they had completed surgery (mastectomy or wide local excision; lymph node clearance or either four-node axillary sampling or sentinel lymph node biopsy), chemotherapy, and radiotherapy (breast and/or axilla), did not have recurrence, and could complete questionnaires in English. Consenting participants completed questionnaires in the waiting room and objective tests in a private clinic room. Medical records were reviewed in order to obtain treatment characteristics. The data used in this secondary analysis included three measures of lymphedema and one of quality of life. 

Quality of life was measured using the Functional Assessment of Cancer Therapy questionnaire with breast cancer and arm function subscales (FACT B+4). There is evidence for its reliability, validity, and practicality [19, 20]. A five-point Likert scale is used, with greater quality of life corresponding with a high score once negatively phrased item scores are reversed. Scores are then calculated by summing the subscale scores, providing physical well-being (PWB), social well-being (SWB), emotional well-being (EWB) functional well-being (FWB), and breast cancer additional concerns subscales (BCC), plus the sum of four questions relating to upper limb swelling and function (arm-specific subscale: AS). All can be interpreted independently [21], and the Trial Outcome Index (TOI) is the sum of the PWB, FWB, and BCC subscales, found to be a better summary index for physical and functional outcomes [19]. Where items are missing for less than 50% of a subscale, the remaining item responses are prorated by using the mean of the answers provided for that subscale [19]. Overall, it is expected that an item response rate of over 80% for the FACT G is achieved (sum of PWB, SWB, EWB, and FWB). 

Perometer (optoelectronic) measurement was used to provide objective measurement of percentage difference in upper limb volume (%LVD) between affected and unaffected limbs. The vertical perometer (400 T) was used; there is evidence for its validity and reliability in populations of women after breast cancer and with known lymphedema [22, 23]. A standardised protocol was used to enhance reliability (Bulley et al., unpublished data). The mean of three measurements for each limb was used where available; the mean of two measurements (in 16 cases) or use of one measurement (in 3 cases) per limb was used if necessary. Where the %LVD was 10% or greater, lymphedema was identified [11]. 

The subjective self-report Morbidity Screening Tool (MST) was developed by the research group. This tool includes a short form focusing on lymphedema; the first question establishes whether or not a person perceives that they have lymphedema (“yes” response), and subsequent questions explore self-reported impacts on activities and participation. The research team investigated its validity and found evidence to support its use; further detail is available elsewhere (Bulley et al., unpublished data). When focusing on the initial question relating to presence or absence of lymphedema, significantly greater %LVD (*n* = 434), FACT G scores, and FACT B+4 arm-specific subscale scores (*n* = 613) were found in those self-reporting lymphedema on the MST, versus those who did not, in those with unilateral treatment (%LVD: *U* = 112128; *p* < 0.001; FACT G: *U* = 14617.0; *p* < 0.001; FACT B+4 arm subscale: *U* = 9671.5; *p* < 0.001). 

The second subjective measure was provided by the Lymphedema and Breast Cancer Questionnaire (LBCQ), a structured interview tool that evaluates 19 symptoms both currently and in the past [1]. Face and content validity and test-retest reliability have been supported and logistic regression found two items to be the best predictors of lymphedema (limb circumference difference of 2 cm or more): “heaviness in the past” and “swelling now” [1]. In the current study, affirming one or both of these items was used as a criterion for identifying or classifying lymphedema. If a participant answered only one of the two items and negated it, the presence of lymphedema according to the LBCQ could not be established and the participant was excluded from analysis.

### 2.3. Analysis

Data were stored in an Access database; SPSS was used to perform descriptive and inferential analysis. Descriptive analysis was conducted using frequencies and percentages where variables were categorical; means and standard deviations for normally distributed continuous data and medians and ranges for nonnormally distributed data. Normality was tested using the Kolmogorov-Smirnov test. In all inferential analysis, tests were two sided and statistical significance was set at *p* < 0.05. 

All available data for each tool were used when determining prevalence estimates for each tool. When analyzing the MST, nonrespondents to questions relating to lymphedema were excluded from analysis, leaving 577 participants. Where a participant had not answered one or both of the LBCQ questions used to identify lymphedema, they were excluded from analysis, leaving 410 people with available data. Bilateral perometry data were available for 389 women.

The three lymphedema prevalence estimates were compared using Cochran's *Q* Test in 341 women who had complete data for all three measures [24]. The proportion of individuals who were identified as having lymphedema by each of the three tools was identified and the Kappa measure of agreement was used to investigate the consistency of lymphedema identification. This compared two tools at a time. Sensitivity and specificity of the two subjective measures were investigated using the objective classification or diagnostic criterion of 10% or greater LVD as a comparator. The subjective tools were compared, using the more established LBCQ as the criterion. 

Analysis of the differences in quality of life between women with and without lymphedema, according to each measurement tool, was conducted in women with complete data for both variables. This was possible for 358 women when analyzing perometry %LVD scores, 378 for the LBCQ and 459 for the MST. Comparisons were conducted using *t* tests (two sided) where data were continuous and normally distributed and the Mann-Whitney *U* test where data were nonnormally distributed. The Chi-square test was used where variables were categorical. 

## 3. Results

Participant characteristics for each dataset are provided in Table 1 and demonstrate similarities among the available data for each measurement tool. To give an indication of the time periods over which treatments were carried out, data were collected between November 2009 and May 2010, at which point 93% of participants were within 10 years after treatment (treatment between May 2000 and November 2009) and 99% were within 15 years after treatment (treatment between May 1995 and November 2009). 

Table 2 summarizes prevalence of lymphedema, which varied from 20.5% to 26.2%, with objective measurement achieving the highest estimate and the MST achieving the lowest. When considering all those responding positively to either or both of the subjective tools, prevalence was similar to the objective estimate. When comparing frequencies of individuals identified as having lymphedema between the measures, no significant difference was demonstrated (Cochran's *Q* = 1.504, *p* = 0.471).

Consistency of identification of lymphedema between measures was evaluated using the Kappa measure of agreement between measurement pairs: a Kappa of 0.207 (*p* < 0.001) resulted between perometry and LBCQ; 0.143 (*p* = 0.008) between perometry and the MST; and 0.531 (*p* < 0.001) between the LBCQ and MST. This suggests moderate agreement between the subjective tools, but poor agreement between each subjective tool and perometry [24].

When evaluating sensitivity as the proportion of correctly identified true positives and specificity as the proportion of correctly identified true negatives [25], perometry %LVD was used as the reference method. The LBCQ had 40.7% sensitivity, compared to 36.8% for the MST—very similar. Specificity was also similar for both subjective tools: 80.0% and 78.1%, respectively. However, it is important to note that less than 50% of lymphedema cases that were objectively identified were also subjectively identified. About a fifth of those without lymphedema according to perometry were found to have lymphedema subjectively. Sensitivity and specificity were found to be 69.0% and 88.2%, respectively when comparing the MST to the LBCQ, higher between subjective tools than when either was compared with perometry. 

When comparing quality of life subscales and the FACT B+4 TOI between those with and without lymphedema for each measurement tool (Table 3), perometry classification did not demonstrate significant differences in all except the arm subscale of the FACT B+4, which includes a question about arm swelling. The subjective measures demonstrated similar results to one another, with significant differences in all FACT B+4 subscales and the TOI for the LBCQ and all except SWB and EWB for the MST. Overall, where significant differences exist in the sample, they appear to be the strongest for the physical and functional subscales and the breast cancer and arm-specific subscales. 

To summarize, objective measurement provided the highest prevalence estimate and the MST the lowest, although no statistically significant differences were found. Poor agreement between methods was found between subjective tools and objective ones, while moderate agreement was found between subjective tools. Sensitivity of the subjective tools, compared with objective, was not high, while specificity was better. Quality of life subscale scores did not differentiate significantly between those with and without lymphedema when using objective classification but did when using either subjective tool. 

## 4. Discussion

The variability in prevalence estimated using the three systems of measuring and classifying lymphedema is consistent with previous results in this area [10, 11, 26], although the relatively small and nonsignificant differences between estimates are unexpected, with a range of only 5.7%. They are less variable than results found in previous studies that compare systems of measuring and classifying lymphedema within single samples. One study used perometry with two diagnostic criteria: 200 mL and 10% or greater change; prevalence estimates at 12 months were 42% and 21%, respectively [11]. Circumferential measurements were used, with a classification criterion of 2 cm increase at a single point, giving an estimate of 70% prevalence. Lastly, the Lymphedema and Breast Cancer Questionnaire [1], which diagnoses lymphedema if participants report signs and symptoms of heaviness and swelling, gave a prevalence estimate of 40%. A further study, with prevalence estimated at 2.5 years after treatment, found that the same criterion gave the highest estimate (2 cm difference in circumferences: 91%), but self-report gave the lowest estimate at 41% [15]. Estimates of 67% and 45% were found for 200 mL change in limb volume (perometry) and 10% or greater limb volume change (perometry), respectively. However, further evidence of greater prevalence estimation by self-report (27.9%) was also found when compared with 11.9% for 5 cm or greater difference in summed arm circumference, 0.6% for 10% or greater summed arm circumference, and 11.4% according to multifrequency bioimpedance, using a difference of 3 or more standard deviations above the reference score [10]. 

There is inconsistency in the existing literature as to whether subjective or objective measures provide higher prevalence estimates [10, 11, 15]. In the current study, objective estimation was found to give the highest prevalence. No other study has been located that compared two subjective measures; it is noticeable that the MST, which utilizes a single question relating to the presence or absence of lymphedema, gave a lower prevalence estimate than the LBCQ, where classification of lymphedema was made where a person affirmed one or both of two separate items. Both included a question relating to swelling, but only the LBCQ also included alterations in the sensation of heaviness when classifying lymphedema. Furthermore, the MST focuses on self-report of sensations “at the moment (e.g., in the past week),” while the LBCQ also addresses “in the past.” One could argue that the latter would include people with swelling that has resolved, which may not reflect chronicity. Some participants were positively diagnosed with lymphedema according to the MST, but not the LBCQ; this may relate to the “cues” provided by the MST question (e.g., tight-fitting rings or clothes), which are not provided in the specific items of “swelling” and “heaviness” within the LBCQ. The two subjective tools appear to have different advantages, with the LBCQ requesting sensations of both swelling and heaviness, while the MST provides cues to aid consideration of the question. If those responding yes to either tool are combined, the subjectively assessed prevalence estimate reaches 27.2%, only 1% greater than the objectively determined estimate. Both choice of items and the way the items are phrased should be carefully considered in subjective classification of lymphedema.

When considering the second research question of whether the three different measurement tools identify the same subsample of subjects as having lymphedema, analysis demonstrated that the three tools identified different subgroups. Statistical analysis demonstrated little consistency between perometry and either of the questionnaires and only moderate agreement between the questionnaires. There have been suggestions previously that self-report does not accurately reflect the presence of lymphedema [27]; however, others believe that subjective assessments can identify early indicators of lymphedema before objectively identified changes occur [5, 28]. In response to this, a latent or subclinical stage has been added to the classification of lymphedema by the International Society of Lymphology [29].

When exploring sensitivity and specificity of lymphedema diagnosis, with the objective measure used as a reference, a relatively high percentage of potentially “positive” cases were not detected by either the LBCQ or MST, while these tools also identified individuals as having lymphedema who were not detected by objective testing. A previous study also reported that when compared with multifrequency electrical impedance, self-report was found to have a sensitivity of 65% but demonstrated an unacceptable number of “false negatives” and “false positives” [10]. Low sensitivity is concerning in relation to early and appropriate intervention, while low specificity could result in inappropriate resource usage and unnecessary anxiety for patients. In the current study, the former is of the greatest concern. 

The differences between objective and subjective tools may reflect measurement of different aspects of a multifaceted condition—physical, cognitive, and affective [1]. Therefore it may not be meaningful to compare objective and subjective tools, if they measure different dimensions. Instead, it may be more valuable to consider which method is best suited for early detection of lymphedema to enable timely intervention and which is best for monitoring changes in the condition in response to treatment. The latter may depend on whether improvement in the condition is best reflected by reduction in swelling, improvement in function, or adaptation in coping. As there is no clear evidence that the amount of swelling correlates with the amount of distress, it may be more valuable to monitor subjective experiences of the condition over time [5, 17]. This is supported by the study finding that quality of life scores differed significantly between those subjectively classified as having or not having lymphedema. This was not evident for objective classification, which is inconsistent with the existing literature that found significant differences in quality of life scores between those objectively identified as having or not having lymphedema [17, 18]. Subjective tools may provide a better reflection of the diversity of physical, functional, social, and psychological symptoms and more strongly reflect the negative relationship between quality of life and lymphedema.

This study made use of a single objective measure and classification of lymphedema, which as previously has been identified to be conservative [11]; future work may benefit from the inclusion of more than one objective system of measuring and classifying lymphedema. It is also important to note that a limitation of this cross-sectional study was that objective measurement and classification of lymphedema relied on bilateral limb comparisons, rather than changes in limb volume over time. This means that normal bilateral asymmetries were not accounted for [1]. 

When arriving at decisions relating to appropriate tools for identifying and monitoring lymphedema over time, it may be beneficial to combine approaches, as suggested previously [1]. Tracking limb volume may give useful information about the efficacy of specific interventions that focus on reducing and maintaining limb volume. Meanwhile, subjective symptom assessment may allow identification of early symptoms and timely referral for management. 

## 5. Conclusion

In this study between one in four and one in five women developed lymphedema after breast cancer treatment. Objective measurement was found to provide higher prevalence estimates than either of the two subjective tools, although no statistically significant differences were found. Poor agreement between methods was found between subjective tools and objective ones, while moderate agreement was found between subjective tools. Sensitivity of the subjective tools, compared with objective ones, was not high, while specificity was better. Quality of life subscale scores did not differentiate significantly between those with and without lymphedema when using objective classification, but did when using either subjective tool. The results support previous suggestions in the literature that lymphedema is multifaceted and that objective tools focus on the physical, while subjective tools reflect the functional and emotional dimensions. This supports the use of both objective and subjective tools in determining early signs of lymphedema development and in monitoring different dimensions of the experience following rehabilitation interventions. Further research is needed to establish an ideal battery of measures that would enable future comparisons between prevalence estimates and studies of treatment efficacy. 

## Figures and Tables

**Table 1:** Participant characteristics.

Participant characteristics (*N* = number)	MST^a^ (*N* = 577)	LBCQ^b^ (*N* = 410)	Perometry (*N* = 389)	Complete data for MST^a^, LBCQ^b^, and perometry (*N* = 341)
Age: mean (SD^c^)	62.21 (10.03)	60.99 (9.97)	60.97 (9.95)	60.49 (9.99)
Months since treatment: median (range)	54 (0–360) (*N* = 575 with data)	50 (0–360) (*N* = 409 with data)	51 (0–360) (*N* = 387 with data)	49 (0–360) (*N* = 341 with data)
Wide local excision: *N* (%^d^)	412 (72.3% of 570)	292 (72.0% of 406)	269 (69.9% of 385)	238 (70.4% of 338)
Mastectomy: *N* (%^d^)	156 (27.4% of 568)	112 (27.8% of 404)	114 (29.8% of 384)	100 (29.6% of 337)
Axillary surgery^e^: *N* (%^d^)	292 (59.3% of 493)	218 (61.5% of 354)	231 (59.6% of 387)	179 (60.4% of 297)
Radiotherapy to the breast: *N* (%^d^)	474 (82.5% of 575)	336 (82.2% of 409)	317 (81.7% of 388)	278 (81.8% of 340)
Radiotherapy to the axilla: *N* (%^d^)	139 (24.3% of 574)	102 (25.1% of 408)	94 (24.4% of 387)	81 (23.8% of 339)

^a^MST: Morbidity Screening Tool.

^b^LBCQ: Lymphedema and Breast Cancer Questionnaire.

^c^SD: standard deviation.

^d^% frequency of number of participants with data for participant characteristic.

^e^Axillary surgery includes lymph node clearance, four-node sampling, and sentinel lymph node biopsy.

**Table 2:** Prevalence of lymphedema according to each measurement tool.

Method of measurement	Prevalence: all available data for each tool	
	Number	% frequency
Perometry	389	26.2
LBCQ^a^	410	23.9
MST^b^	577	20.5
Subjective (yes to either LBCQ^a^/MST^b^/both)	577	27.2

^a^MST: Morbidity Screening Tool.

^b^LBCQ: Lymphedema and Breast Cancer Questionnaire.

**Table 3:** Differences in quality of life in those with and without lymphedema according to each measurement tool.

FACT B+4^a^ subscales	No lymphedema: median	Lymphedema: median	Inferential analysis of difference:	
			the Mann-Whitney *U* test	
			*Z* value	*p* value
Analysis using Perometry data				
TOI^b^ (n = 357)	94	92	−0.829	0.407
PWB^c^ (N = 359)	26	26	−0.623	0.533
SWB^d^ (N = 356)	26	26	−0.329	0.742
EWB^e^ (N = 357)	21	21	−0.536	0.592
FWB^f ^(N = 359)	25	25	−0.151	0.880
BCC^g^ (N = 357)	43	42	−1.64	0.100
AS^h^ (N = 357)	20	18	−2.96	0.003*

Analysis using LBCQ^i^ data				
TOI^b^ (n = 376)	95	84	−6.746	**<**0.005*
PWB^c^ (N = 378)	27	24	−6.158	**<**0.005*
SWB^d^ (N = 376)	27	24.5	−3.426	0.001*
EWB^e^ (N = 376)	21	20	−2.845	0.004*
FWB^ f^ (N = 378)	25	22.5	−4.370	**<**0.005*
BCC^g^ (N = 376)	44	36	−7.449	**<**0.005*
AS^h^ (N = 376)	20	15	−9.774	**<**0.005*

Analysis using MST^j^ data				
TOI^b^ (n = 453)	94	84.5	−6.445	**<**0.005*
PWB^c^ (N = 459)	26	24	−5.004	**<**0.005*
SWB^d^ (N = 454)	26	26	−1.234	0.217
EWB^e^ (N = 456)	21	20	−1.510	0.131
FWB^ f^ (N = 459)	25	22.5	−3.382	0.001*
BCC^g^ (N = 453)	44	37	−7.698	**<**0.005*
AS^h^ (N = 453)	20	15	−10.279	**<**0.005*

^a^FACT B+4: Functional Assessment of Cancer Therapy-Breast Cancer subscale.

^b^TOI: Trial Outcome Index.

^c^PWB: physical well-being.

^d^SWB: social well-being.

^e^EWB: emotional well-being.

^f^FWB: functional well-being.

^g^BCC: breast cancer specific concerns.

^h^AS: arm-specific subscale.

^i^MST: Morbidity Screening Tool.

^j^LBCQ: Lymphedema and Breast Cancer Questionnaire.

*p value is statistically significant.
